# Antimicrobial susceptibility pattern of clinical isolates of *Burkholderia pseudomallei* in Bangladesh

**DOI:** 10.1186/s13104-017-2626-5

**Published:** 2017-07-20

**Authors:** Subarna Dutta, Sabah Haq, Mohammad Rokibul Hasan, Jalaluddin Ashraful Haq

**Affiliations:** 1 Department of Microbiology, Bangladesh Institute of Research and Rehabilitation in Diabetes, Endocrine and Metabolic Disorders, 122 Kazi Nazrul Islam Avenue, Dhaka, 1000 Bangladesh; 2 Department of Microbiology, Ibrahim Medical College, 122 Kazi Nazrul Islam Avenue, Dhaka, 1000 Bangladesh

**Keywords:** *Burkholderia pseudomallei*, Antimicrobial susceptibility, Bangladesh, Minimum inhibitory concentration

## Abstract

**Background:**

Melioidosis an infectious disease, caused by a Gram negative bacterium called *Burkholderia pseudomallei*, is endemic in Bangladesh. This organism is sensitive to limited number of antimicrobial agents and need prolonged treatment. There is no comprehensive data on the antimicrobial susceptibility profile of *B. pseudomallei* isolated in Bangladesh over last several years. The present study aimed to determine the antimicrobial susceptibility pattern of *B. pseudomallei* isolated in a tertiary care hospital of Dhaka city from 2009 to 2015.

**Methods:**

All *B. pseudomallei* isolated from melioidosis patients over a period of 7 years (2009–2015) in the Department of Microbiology of a 725-bed tertiary care referral hospital in Dhaka city, Bangladesh were included in the study. *B. pseudomallei* was identified by Gram stain, culture, specific biochemical tests, serology and PCR using specific primers constructed from 16s rRNA region of *B. pseudomallei*. Antimicrobial susceptibility to specific agents was determined by disk diffusion and minimum inhibitory concentration methods.

**Results:**

A total of 20 isolates of *B. pseudomallei* which were isolated from patients coming from different geographic locations of Bangladesh were included in the study. All the isolates were uniformly sensitive (100%) to ceftazidime, imipenem, piperacillin–tazobactam, amoxicillin–clavulanic acid and tetracycline by both disk diffusion and MIC methods. Two strains were resistant to trimethoprim–sulfamethoxazole by disk diffusion method but were sensitive by MIC method. The MIC_50_ and MIC_90_ values of the above antimicrobial agents were almost similar. All the isolates were resistant to amikacin by both MIC and disk diffusion methods.

**Conclusion:**

The results of the study suggest that *B. pseudomallei* prevalent in Bangladesh were still susceptible to all recommended antimicrobial agents used for the treatment of melioidosis. However, regular monitoring is needed to detect any emergence of resistance and shifting of MIC_50_ and MIC_90_ values.

## Background

*Burkholderia pseudomallei* is a Gram negative saprophytic bacterium that causes melioidosis. It is an endemic disease of public health and clinical importance in many countries of the world including Bangladesh [1–3]. Several cases have been reported from Bangladesh since 1988 [3, 4]. Recently, we have found that the organism is present in the soil samples of Bangladesh and about 22.6–30.8% of people residing in melioidosis endemic districts of Bangladesh were sero-positive for anti- *B. pseudomallei* IgG antibody [5].

Melioidosis may be manifested as localized or disseminated infection involving multiple organs of the body. The disease is usually fatal if left untreated. Treatment of melioidosis requires prolonged courses of antibiotics. The current treatment regime has an intensive and maintenance phase. In the intensive phase, ceftazidime is usually the drug of choice. However, carbapenem (imipenem/meropenem) is recommended in severe infection and treatment failure cases. This is followed by the maintenance phase which consists of oral administration of TMP–SMX or TMP–SMX and doxycycline or amoxicillin–clavulanic acid [6–9]. But recently, there are reports of resistant *B. pseudomallei* to ceftazidime, TMP–SMX, amoxicillin–clavulanic acid from different melioidosis endemic countries of the world including neighboring India [9–15].

Currently, no comprehensive information is available on the antimicrobial susceptibility pattern of *B. pseudomallei* isolated from patients in Bangladesh. Therefore, the present study was undertaken to determine the antibiotic susceptibility pattern of *B. pseudomallei* isolated from patients during the period of 2009–2015 in our hospital by disc diffusion and minimum inhibitory concentration (MIC) methods.

## Methods

### Study organisms

All *B. pseudomallei* isolated from melioidosis patients over a period of 7 years (January 2009 to December 2015) in the Department of Microbiology of a 725-bed tertiary care referral hospital in Dhaka city, Bangladesh were included in the study. *B. pseudomallei* was identified by typical colony morphology, Gram staining (bipolar staining), motility, biochemical tests, arabinose assimilation and resistance to colistin and aminoglycoside [16]. Monoclonal antibody based latex agglutination test (Melioidosis Research Center, Khon Kaen, Thailand) was performed for the final identification and confirmation of *B. pseudomallei*. Serologically confirmed *B. pseudomallei* isolates were further confirmed by PCR using specific primers constructed from 16s rRNA region of *B. pseudomallei* [17].

### Determination of MIC

Minimum inhibitory concentrations of all antibiotics against *B. pseudomallei* were determined by agar dilution method [18]. Briefly, Muller–Hinton agar plates containing different dilutions of specific antimicrobial agent were spot inoculated with 10^4^ organisms. Each spot was 5–8 mm in diameter. All reading was taken between 18 and 24 h of incubation at 37 °C in aerobic condition. The MIC represented the lowest concentration of antimicrobial agents at which complete inhibition of growth of the organism occurred. The MIC (μg/ml) interpretation for susceptible (S), intermediate (I), and resistant (R) for amoxicillin–clavulanic acid, ceftazidime, imipenem, tetracycline and TMP–SMX was carried out following the CLSI approved guideline M45-A2 [19]. As reported earlier, the MIC breakpoint of *P. aeruginosa* for piperacillin–tazobactam, amikacin and gentamicin as mentioned in CLSI guideline was used to interpret the MIC of *B. pseudomallei* [14, 20]. The detail MIC interpretive values used in the study are shown in Table 1.

**Table 1:** Zone diameter interpretive standards and MIC breakpoints for *B. pseudomallei* used in the study

Antimicrobial agents	Disk diffusion test				MIC (µg/ml)		
	Disk content (µg)	Zone diameter (mm)					
		R	I	S	S	I	R
Ceftazidime^a, b^	30	≤14	15–16	≥18	≤8	16	≥32
Imipenem^a, b^	10	≤15	16–18	≥19	≤4	8	≥16
Amox–Clav^a,d^	20/10	≤13	14–17	≥18	≤8/4	16/8	≥32/16
Pip–Tazo^b, c^	100/10	≤14	15–20	≥21	≤16/4	32/4–64/4	≥128/4
TMP–SMX^a, d^	1.25/23.75	≤10	11–15	≥16	≤2/38	–	≥4/76
Tetracycline^a, d^	30	≤11	12–14	≥15	≤4	8	≥16
Amikacin^b, c^	30	≤14	15–16	≥17	≤16	32	≥64
Gentamicin^b, c^	10	≤12	13–14	≥15	≤4	8	≥16

*TMP*–*SMX* trimethoprim/sulfamethoxazole, *Pip*–*Tazo* piperacillin–tazobactam, *Amox*–*Clav* amoxicillin/clavulanic acid, *MIC* minimum inhibitory concentration

^a^MIC interpretive standards defined in M45-A2, CLSI guideline for *B. pseudomallei* [19]

^b^Zone diameter interpretive standards of CLSI for *P. aeruginosa* by disk diffusion method [20]

^c^MIC interpretive standard of CLSI for *P. aeruginosa* [20]

^d^Zone diameter interpretive standards of CLSI for *Enterobacteriaceae* by disk diffusion method [20]

### Disc diffusion method

The isolates were tested for susceptibility to ceftazidime (30 µg), imipenem (10 µg) piperacillin–tazobactam (30 µg), amoxicillin–clavulanic acid (30 µg), tetracycline, TMP–SMX (1.25/23.75 µg), gentamicin (10 µg) and amikacin (30 µg) by disc diffusion technique [21]. Briefly, a 0.5 McFarland suspension of each bacterial isolate was made in normal saline and inoculated onto Mueller–Hinton agar (Oxoid Ltd, UK) to prepare a uniform lawn. The disks were applied at a specific distance from each other and the zone of inhibition around the antibiotic disc was measured after 18 h of incubation of plates at 37 °C. Since interpretative standards for zone sizes of tested antibiotics are not available for *B. pseudomallei*, we used the Clinical and Laboratory Standards Institute [20] threshold zone sizes for members of the family *Enterobacteriaceae* and *Pseudomonas aeruginosa* as reported earlier [14]. The zone of inhibition was interpreted as sensitive, intermediate and resistant according to CLSI guideline. The zone sizes used for interpretation are shown in Table 1.

Each isolate was tested in duplicate by disk diffusion and MIC methods. All disks and antibiotics were obtained from Oxoid Ltd., Basingstoke, Hampshire, UK and Sigma, USA. Potency of the disks and antimicrobial agents were standardized using the reference strain *E. coli* ATCC 25922.

## Results

A total of 20 *B. pseudomallei* which were isolated from patients coming from different geographic locations of Bangladesh were included in the study. All the isolates were uniformly sensitive (100%) to ceftazidime, imipenem, piperacillin–tazobactam, amoxicillin–clavulanic acid and tetracycline by both disk diffusion and MIC methods. Two strains were resistant to TMP–SMX by disk diffusion method but were sensitive by MIC method (Table 2). The MIC value of those two isolates were 1/19 and 2/38 µg/ml while by disk diffusion test their zone diameter was below the recommended cut off value of 16 mm. The MIC_50_ and MIC_90_ values of the above antimicrobial agents were almost similar (Table 2). The MIC_50_ and MIC_90_ of amoxicillin/clavulanic acid were towards higher side compared to other agents tested. All the isolates were resistant to amikacin by both MIC and disk diffusion methods. All 20 isolates were resistant to gentamicin by disk diffusion test.

**Table 2:** Results of disk diffusion and MIC tests of *B. pseudomallei* isolated during 2009–2015 (N = 20)

Antimicrobial agents	Disk diffusion test			MIC test		
	Disk content (µg)	Sensitive N (%)	Zone diameter range (mm)	Sensitive N (%)	MIC_50_ (µg/ml)	MIC_90_ (µg/ml)
Ceftazidime	30	20 (100)	24–32	20 (100)	2	2
Imipenem	10	20 (100)	33–40	20 (100)	2	2
Amox–Clav	20/10	20 (100)	19–28	20 (100)	8/4	8/4
Pip–Tazo	100/10	20 (100)	35–39	20 (100)	2/4	2/4
TMP–SMX	1.25/23.75	18 (90)	15–28	20 (100)	1/19	2/38
Tetracycline	30	20 (100)	17–31	20 (100)	0.5	1
Amikacin	30	0 (0)	10–15	0 (0)	64	64
Gentamicin	10	0 (0)	0	ND	ND	ND

*TMP*–*SMX* trimethoprim/sulfamethoxazole, *Pip*–*Tazo* piperacillin–tazobactam, *Amox*–Clav amoxicillin/clavulanic acid, *MIC* minimum inhibitory concentration, *ND* not done

## Discussion

Mortality in patients with *B. pseudomallei* infection is high and early diagnosis and specific antimicrobial therapy is necessary to reduce the fatal outcome [21–23]. The antibiotic of choice for melioidosis is ceftazidime, a third generation cephalosporin. Imipenem is considered as alternatives to ceftazidime. Since relapse rates are high, initial parenteral treatment followed by maintenance therapy with oral TMP–SMX (8–12 & 40–62 mg/kg/day) or doxycycline (4 mg/kg/day) or amoxicillin–clavulanic acid (20/5 mg/kg 8 h) for 12–20 weeks are recommended [6–8]. However, *B. pseudomallei* resistant to ceftazidime, tetracycline/doxycycline and TMP–SMX, has been recently reported from Malaysia, Thailand and Australia. About 0.6% *B. pseudomallei* in Malaysia was found to be resistant to imipenem, ceftazidime, doxycycline and amoxicillin–clavulanic acid while the rate was 10% for TMP–SMX [9]. The rate was around 0.6% in Thailand [14]. About 4% *B. pseudomallei* in Australia was reported as resistant to imipenem, ceftazidime, doxycycline and TMP–SMX [12].

In Bangladesh there was no comprehensive data available on the antibiotic susceptibility pattern of *B. pseudomallei*. Bangladesh is a melioidosis endemic country and several cases have been documented since 1988 and, recently we have reported its presence in the soil from a district where several cases of melioidosis were diagnosed [3–5]. Organisms included in the present study were isolated from patients mainly coming from different areas of north and north-eastern part of Bangladesh during the period of 2009–2015. The result showed that all the isolates were uniformly sensitive to imipenem, ceftazidime, piperacillin–tazobactam, tetracycline, amoxicillin–clavulanic acid and TMP–SMX. The absence of resistance to these antibiotics in our isolates might be due to the fact that only a few numbers of organisms were tested. Therefore, in our settings, these antimicrobial agents can empirically be used to treat suspected melioidosis patients prior to the availability of culture and sensitivity results.

Comparing the results of the disk diffusion test with that of MIC it appeared that the CLSI recommended breakpoint of zone diameter for *P. aeruginosa*, and *Enterobacteriaceae* could be used to interpret the zone of inhibition by *B. pseudomallei* in disk diffusion test where facilities for MIC test is lacking or till the zone diameter of *B. pseudomallei* for these agents are standardized. MIC and disk diffusion tests showed concordant results for all antimicrobials except for TMP–SMX. Only, two isolates were resistant to TMP–SMX by disk diffusion method (zone diameter 15 mm) whereas both were found sensitive to TMP–SMX by MIC method (≤1/19 and 2/38 µg/ml). Since MIC method is considered as the gold standard for determining the susceptibility of *B. pseudomallei* to TMP–SMX [19], therefore, all our 20 isolates were actually sensitive to TMP–SMX. The interpretation of susceptibility of *B. Pseudomallei* to TMP–SMX by disk diffusion test is sometimes affected by slight hazy growth within the zone of inhibition and blurring of margin. Therefore, the doubtful result of disk diffusion test for TMP–SMX should be confirmed by MIC method.

*Burkholderia pseudomallei* is intrinsically resistant to aminoglycosides like gentamicin and amikacin [24]. These antibiotics are used as diagnostic agents for identification of *B. pseudomallei* in culture. Also, most selective media incorporate gentamicin in the medium as a selective agent to isolate *B. pseudomallei* from soil and other samples. Though *B. pseudomallei* is said to be resistant to aminoglycosides, there have been reports of aminoglycoside sensitive isolates (0.1%) from Thailand [25, 26], and from Australia [27]. Recently it has been reported that 86% of *B. pseudomallei* isolates in Sarawak, Malaysian Borneo were sensitive to gentamicin [28]. The strains belonged to sequence type 881 and 997. In view of the above, we have determined the susceptibility of our strains to amikacin, an aminoglycoside. All the 20 isolates were resistant to amikacin by both disk diffusion (zone diameter range 10–15 mm) and MIC method (MIC 64–512 µg/ml). It may be mentioned that all these strains were also resistant to gentamicin where no zone of inhibition was observed by disk diffusion method. Therefore, it is assumed that amikacin and gentamicin may be used safely as a diagnostic disk and in selective media for isolation of the organism from soil and other contaminated samples.

## Conclusion

The study, though with limited number of isolates, revealed that the prevalent *B. pseudomallei* in Bangladesh were susceptible to recommended antimicrobial agents used for both intensive and maintenance phase therapy. Continuous monitoring of antimicrobial susceptibility should be maintained to detect emergence of resistant organism.

## Abbreviations

TMP–SMX: trimethoprim–sulfamethoxazole
Pip–Tazo: piperacillin–tazobactam
Amox–Clav: amoxicillin/clavulanic acid
PCR: polymerase chain reaction
MIC: minimum inhibitory concentration
IgG: immunoglobulin G
CLSI: Clinical and Laboratory Standards Institute

## References

[1] Limmathurotsakul D, Dance DAB, Wuthiekanun V, Kaestli M, Mayo M, Warner J (2013). Systematic review and consensus guidelines for environmental sampling of *Burkholderia pseudomallei*. PLoS Negl Trop Dis.

[2] Limmathurotsakul D, Peacock SJ (2011). Melioidosis: a clinical overview. Br Med Bull.

[3] Barai L, Jilani MSA, Haq JA (2014). Melioidosis—case reports and review of cases recorded among Bangladeshi population from 1988–2014. Ibrahim Med College J.

[4] Struelens MJ, Mondol G, Bennish M, Dance DA (1988). Melioidosis in Bangladesh: a case report. Trans R Soc Trop Med Hyg.

[5] Jilani MSA, Robayet JAM, Mohiuddin M, Hasan MR, Ahsan CR, Haq JA (2016). *Burkholderia pseudomallei*: its detection in soil and seroprevalence in Bangladesh. PLoS Negl Trop Dis.

[6] Samuel M, Ti TY (2002). Interventions for treating melioidosis. Cochrane Database Syst Rev.

[7] Pitman MC, Luck T, Marshall CS, Anstey NM, Ward L, Currie BJ (2015). Intravenous therapy duration and outcomes in melioidosis: a new treatment paradigm. PLoS Negl Trop Dis.

[8] Gilad J, Harary I, Dushnitsky T, Schwartz D, Amsalem Y (2007). *Burkholderia mallei* and *Burkholderia pseudomallei* as bioterrorism agents: national aspects of emergency preparedness. Israel Med Assoc J.

[9] Ahmad N, Hashim R, Noor AM (2013). The in vitro antibiotic susceptibility of Malaysian isolates of *Burkholderia pseudomallei*. Int J Microb.

[10] Irmawanti-Rahayu S, Noorhamdani AS, Santoso S (2014). Resistance pattern of *Burkholderia pseudomallei* from clinical isolates at Dr. Saiful Anwar general hospital, Malang-Indonesia. J Clin Microbiol Infect Dis.

[11] Sarovich DS, Price EP, Von Schulze AT, Cook JM, Mayo M, Watson LM, Richardson L, Seymour ML, Tuanyok A, Engelthaler DM, Pearson T, Peacock SJ, Currie BJ, Keim P, Wagner DM (2012). Characterization of ceftazidime resistance mechanisms in clinical isolates of *Burkholderia pseudomallei* from Australia. PLoS ONE.

[12] Jenny AWJ, Lum G, Fisher DA, Currie BJ (2001). Antibiotic susceptibility of *Burkholderia pseudomallei* from tropical northern Australia and implications for therapy of melioidosis. Int J Antimicrob Agents.

[13] Thibault FM, Hernandez E, Vidal DR, Girardet M, Cavallo JD (2004). Antibiotics susceptibility of 65 isolates of *Burkholderia pseudomallei* and *Burkholderia mallei* to 35 antimicrobial agents. J Antimicrob Chemother.

[14] Wuthiekanun V, Amornchai P, Saiprom N, Chantratita N, Chierakul W, Koh GCKW (2011). Survey of antimicrobial resistance in clinical *Burkholderia pseudomallei* isolates over two decades in northeast Thailand. Antimicrob Agents Chemother.

[15] Behera B, Prasad Babu TL, Kamalesh A, Reddy G (2012). Ceftazidime resistance in *Burkholderia pseudomallei*: first report from India. Asian Pac J Trop Med.

[16] Lipuma JJ, Currie BJ, Lum GD, Vandamme PAR, Murray PR, Baron EJ, Jorgensen JH, Landry ML, Pfaller MA (2007). *Burkholderia*, *Stenotrophomonas*, *Ralstonia*, *Cupriavidus*, *Pandrea*, *Brevundimonus*, *Comamonas*, *Delftia*, and *Acidovorax*. Manual of clinical microbiology.

[17] Maude R, Maude R, Ghose A, Amin M, Islam M, Ali M (2012). Seroepidemiological surveillance of *B. pseudomallei* in Bangladesh. Trans R Soc Trop Med Hyg.

[18] Washington JA, Lennette EH, Balows A, Hausler WJ, Shadomy HJ (1985). Susceptibility tests: agar dilution. Manual of clinical microbiology.

[19] Clinical and Laboratory Standards Institute (2010). Methods for antimicrobial dilution and disk susceptibility testing of infrequently isolated or fastidious bacteria M45-A2.

[20] Performance Standards for Antimicrobial Susceptibility Testing M100-S25 (2015). Twenty-fifth Informational Supplement.

[21] Bauer AW, Kirby WMM, Sherris JC, Truck M (1966). Antibiotic susceptibility testing by a standardized single disk method. Am J Clin Pathol.

[22] Cheng AC, Currie BJ (2005). Melioidosis: epidemiology, pathophysiology and management. Clin Microbiol Rev.

[23] Haq JA (1998). Melioidosis in Bangladesh. Bangladesh J Pathol.

[24] Moore RA, DeShazer D, Reckseidler S, Weissman A, Woods DE (1999). Efflux-mediated aminoglycoside and macrolide resistance in *Burkholderia pseudomallei*. Antimicrob Agents Chemother.

[25] Simpson AJH, White NJ, Wuthiekanun V (1999). Aminoglycoside and macrolide resistance in *Burkholderia pseudomallei*. Antimicrob Agents Chemother.

[26] Trunck LA, Propst KL, Wuthiekanun V, Tuanyok A, Beckstrom-Sternberg SM, Beckstrom-Sternberg JS, Peacock SJ, Keim P, Dow SW, Schweizer HP (2009). Molecular basis of rare aminoglycoside susceptibility and pathogenesis of *Burkholderia pseudomallei* clinical isolates from Thailand. PLoS Negl Trop Dis.

[27] Price EP, Sarovich DS, Mayo M, Tuanyok A, Drees KP, Kaestli M, Beckstrom-Sternberg SM, Babic-Sternberg JS, Kidd TJ, Bell SC, Keim P, Pearson T, Currie BJ (2013). Within-host evolution of *Burkholderia pseudomallei* over a twelve-year chronic carriage infection. MBio.

[28] Podin Y, Sarovich DS, Price EP, Kaestli M, Mayo M, Hii K (2014). *Burkholderia pseudomallei* isolates from Sarawak, Malaysian Borneo, are predominantly susceptible to aminoglycosides and macrolides. Antimicrob Agents Chemother.

